# mbDriver: identifying driver microbes in microbial communities based on time-series microbiome data

**DOI:** 10.1093/bib/bbae580

**Published:** 2024-11-11

**Authors:** Xiaoxiu Tan, Feng Xue, Chenhong Zhang, Tao Wang

**Affiliations:** Department of Bioinformatics and Biostatistics, School of Life Sciences and Biotechnology, Shanghai Jiao Tong University, 800 Dongchuan Road, Minhang District, Shanghai 200240, China; Department of Bioinformatics and Biostatistics, School of Life Sciences and Biotechnology, Shanghai Jiao Tong University, 800 Dongchuan Road, Minhang District, Shanghai 200240, China; State Key Laboratory of Microbial Metabolism and Ministry of Education Key Laboratory of Systems Biomedicine, School of Life Sciences and Biotechnology, Shanghai Jiao Tong University, 800 Dongchuan Road, Minhang District, Shanghai 200240, China; Department of Bioinformatics and Biostatistics, School of Life Sciences and Biotechnology, Shanghai Jiao Tong University, 800 Dongchuan Road, Minhang District, Shanghai 200240, China; SJTU-Yale Joint Center of Biostatistics and Data Science, Shanghai Jiao Tong University, 800 Dongchuan Road, Minhang District, Shanghai 200240, China; MoE Key Lab of Artificial Intelligence, AI Institute, Shanghai Jiao Tong University, 800 Dongchuan Road, Minhang District, Shanghai 200240, China

**Keywords:** community dynamics, denoising, ecological network, time series abundance data

## Abstract

Alterations in human microbial communities are intricately linked to the onset and progression of diseases. Identifying the key microbes driving these community changes is crucial, as they may serve as valuable biomarkers for disease prevention, diagnosis, and treatment. However, there remains a need for further research to develop effective methods for addressing this critical task. This is primarily because defining the driver microbe requires consideration not only of each microbe’s individual contributions but also their interactions. This paper introduces a novel framework, called mbDriver, for identifying driver microbes based on microbiome abundance data collected at discrete time points. mbDriver comprises three main components: (i) data preprocessing of time-series abundance data using smoothing splines based on the negative binomial distribution, (ii) parameter estimation for the generalized Lotka-Volterra (gLV) model using regularized least squares, and (iii) quantification of each microbe’s contribution to the community’s steady state by manipulating the causal graph implied by gLV equations. The performance of nonparametric spline-based denoising and regularized least squares estimation is comprehensively evaluated on simulated datasets, demonstrating superiority over existing methods. Furthermore, the practical applicability and effectiveness of mbDriver are showcased using a dietary fiber intervention dataset and an ulcerative colitis dataset. Notably, driver microbes identified in the dietary fiber intervention dataset exhibit significant effects on the abundances of short-chain fatty acids, while those identified in the ulcerative colitis dataset show a significant correlation with metabolism-related pathways.

## Introduction

Various conditions and diseases, including obesity, inflammatory bowel disease, and colorectal cancer, have been demonstrated to be linked to alterations in the human gut microbiome [1–4]. The prospect of manipulating the microbiome has opened new doors to innovative therapeutic approaches [5]. For example, fecal microbiota transplantation (FMT) is an emerging therapy that has been successfully used to treat recurrent *Clostridium difficile (C. difficile)* infections [6–10]. FMT involves the transfer of gut microbiota from a healthy individual into the gut of a patient, with the goal of restoring the structure and function of the patient’s gut microbiota to achieve therapeutic effects [11]. During this process, specific transplanted microbes play a role in regulating the growth and proliferation of *C. difficile*.

The above example prompts us to delve into the concept of driver microbes. It is worth noting that various studies may delineate driver microbes differently, e.g. through the comparison of association networks [12] or from the perspective of network control [13, 14]. In this paper, driver microbes are defined as microbes capable of inducing changes in the community’s steady state and contributing significantly to these changes. Specifically, the presence or absence of these microbes can substantially influence the abundance of other species at the community’s steady state, thereby altering the overall structure and function of the community. Much like the unknown regulators in FMT, the identification of driver microbes associated with diseases holds the promise of shedding light on disease mechanisms and presenting potential targets for disease intervention. The experimental validation of driver microbes would usually involve comparing the effects on the community’s structure and function when individual community members are removed and/or added. However, the sheer multitude of microbial species and the presence of unculturable microbes make these experiments difficult [15]. Therefore, the quest to pinpoint driver microbes within microbial communities through experimental methods poses a formidable challenge.

High-throughput omics techniques have been widely used in microbiome research [16]. These technologies have enabled the collection of microbiome data, spanning DNA, RNA, proteins, and metabolites, for comprehensively understanding microbial communities [17, 18]. In particular, leveraging microbiome data allows for the construction of species interaction networks, providing profound insights into microbial ecosystems [19, 20]. These networks, including co-occurrence and ecological networks, visually represent interactions between various microbial species, have found broad utility in microbiome data analysis [9, 21–25]. Co-occurrence networks are often derived from cross-sectional data, highlighting statistical associations among species, whereas ecological networks utilize temporal data to illustrate regulatory relationships among species. It is important to note that identifying driver microbes based on interaction networks crucially depends on defining a network metric capable of assessing the significance of species within a community. Many existing metrics, such as betweenness centrality, hub, and NESH [12, 23, 26], are designed for co-occurrence networks and thus lack biological interpretation. In an attempt to address this limitation, a novel approach known as MDSINE was proposed specifically for ecological networks [27]. However, as we will demonstrate, this approach either underutilizes the data or poses significant computational demands. Consequently, the identification of driver microbes remains a challenging research endeavor.

**Figure 1:**
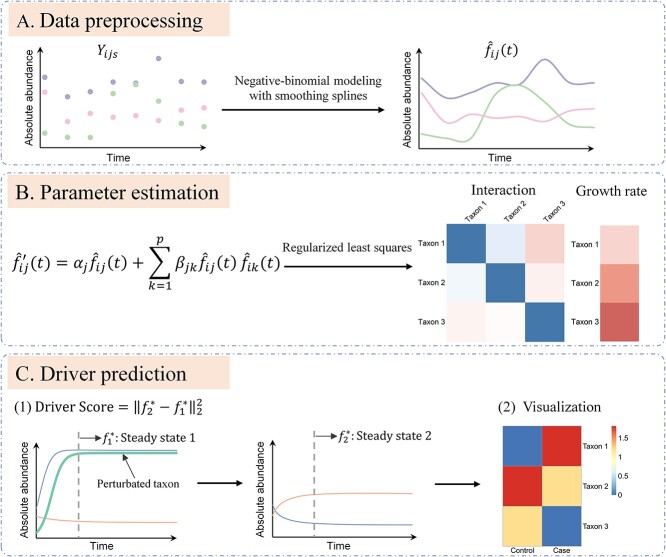
Framework of mbDriver. **A**. Data preprocessing: Smoothing splines are employed to denoise and smooth observed abundance data. **B**. Parameter estimation: Penalized least squares are applied to estimate parameters within the dynamic model. **C**. Driver prediction: Driver scores are calculated, and microbes are ranked based on the magnitude of these scores. A higher driver score for a microbe indicates its capacity to induce more significant changes in the community. The left and middle panel of C illustrate the community’s steady state before and after perturbing a taxon, with species abundances varying over time according to the gLV model.

To address this issue, this paper presents a computational framework, called mbDriver, for identifying driver microbes in microbial communities based on time-series abundance data. As shown in Fig. 1, mbDriver consists of three main components, integrating non-parametric statistics, community dynamics modeling, and graph manipulation, with the following details. **A. Data preprocessing:** Observed temporal abundance data are denoised and smoothed, using smoothing splines based on the negative binomial distribution, to obtain estimates of the species abundance curves and their derivatives. **B. Parameter estimation:** The generalized Lotka-Volterra (gLV) equations and regularized least squares are employed for dynamic modeling and estimation of the growth rates and interaction parameters in the gLV model, respectively. **C. Driver prediction:** The Driver score index is introduced for identifying driver microbes in a microbial community. This index quantifies the impact of each microbe on changes in the community’s steady state, and is derived from manipulating the causal graph implied by the gLV equations. A higher score for a microbe indicates its capability to induce more significant changes in the community. See the **Methods** section for details. Simulated and real-world datasets are used for comprehensively evaluating the effectiveness of mbDriver and for illustrating its application in the prediction of microbiome-based disease treatment targets.

## Methods

### Generalized Lotka-Volterra model

The gLV model, which consists of a set of coupled ordinary differential equations, extends the classical predator–prey (Lotka-Volterra) model, describing how the absolute abundances of a large number of species change over time [28, 29]. mbDriver uses this physical model to describe the interactions between species in a microbial community. Suppose there are $n$ subjects and $p$ species. Let ${f}_{ij}(t)$ denote the absolute abundance of species $j$ in subject $i$ at time $t$, and let ${f}_{ij}^{\prime }(t)$represents the rate of change in ${f}_{ij}(t)$ over time, where $i=1,\dots, n$, $j=1,\dots, p$. Mathematically, the model can be expressed as follows:


(1) \begin{equation*} {f}_{ij}^{\prime }(t)={\alpha}_j{f}_{ij}(t)+{\sum}_{k=1}^p{\beta}_{jk}{f}_{ij}(t){f}_{ik}(t). \end{equation*}

Here, ${\alpha}_j$ is the intrinsic growth rate of species$j$, and ${\beta}_{jk}$ represents the interaction intensity between species $j$ and $k$, for $j,k=1,\dots, p$. In this model, the interacting species might have a wide range of relationships, including competition, cooperation, or neutralism.

The main assumptions of the gLV model are: (i) absolute abundance data is required; (ii) parameters such as growth rates and interactions are assumed to be universal; and (iii) interactions between species are pairwise [28]. Consequently, the gLV model is increasingly used to describe stable microbial communities where interspecies interactions are relatively simple, such as microbial community dynamics under laboratory conditions (e.g. synthetic ecosystems or controlled environments). Its key advantage lies in its simplicity: all parameters can be inferred and interpreted from temporal community data, provided the data is sufficiently informative [30, 31]. One application of the gLV model is to explore the impact that any given microbe has on the abundance of other microbes, such as designing microbiome-based therapeutic approaches (e.g. personalized probiotic cocktails) to achieve a desired microbial composition [9]. Additionally, the gLV model has been applied to host-associated microbial communities, revealing complex interactions among microbes and identifying keystone species [27, 32–34].

It is known that dynamic models, including the gLV model, may lack distinguishability or identifiability. This means that if temporal data are insufficiently informative, different sets of model parameters can yield identical trajectories [31]. However, due to cost and technical constraints, temporal microbiome data are often sparsely sampled or observed at limited intervals. To tackle the identifiability challenge, we assume that the parameters in the gLV model are shared across all individuals within the same population. This assumption becomes unnecessary when data are densely collected over time.

### Data preprocessing

The analysis of microbiome data using gLV equations has several challenges. First, the true underlying biological signals, ${f}_{ij}(t)$ and ${f}_{ij}^{\prime }(t)$, are unknown. Second, due to various experimental and technical factors, the observed data is contaminated with noise, which can obscure biological signals. Third, microbiome data are collected at discrete rather than continuous time points. To address these issues, we need to apply preprocessing techniques to remove or reduce this noise. Denoising helps improve the accuracy and reliability of microbiome data analysis [35].

For subject $i$, suppose the data are collected consecutively at ${m}_i$time points. Let ${Y}_{ijs}$ denote the observed abundance of species $j$ in subject $i$ at the $s$-th time point, where $s=1,\dots, {m}_i$. We assume a negative binomial distribution for ${Y}_{ijs}$:


(2) \begin{equation*} {Y}_{ijs}\sim NB\left({\mu}_{ijs},{\varphi}_j\right), \end{equation*}

where ${\mu}_{ij s}={f}_{ij}\left({t}_s\right)$ is the mean of the negative binomial distribution, and${\varphi}_j$denotes the dispersion parameter. We then use smoothing splines for denoising and smoothing the data separately for each species [36]. The estimation procedure is implemented using the **gam** function from the **mgcv** R package (v1.8–42) [37]. We denote the estimates of species abundance curves and their derivatives by ${\hat{f}}_{ij}(t)$ and ${\hat{f}}_{ij}^{\prime }(t)$, respectively.

Please note that mbDriver is capable of accepting input data in two formats: absolute abundances of microbial taxa, or a combination of relative abundance data and biomass data [38]. In the latter scenario, the relative abundances are converted into absolute numerical values.

### Parameter estimation

We can rewrite Eq. (1) as:


(3) \begin{equation*} \frac{f_{ij}^{\prime }(t)}{f_{ij}(t)}={\alpha}_j+{\sum}_{k=1}^p{\beta}_{jk}{f}_{jk}(t). \end{equation*}

Replacing $f_{ij}(t)$ and $f_{ij}^{\prime}(t)$ by $\hat{f}_{ij}(t)$ and $\hat{f}_{ij}^{\prime}(t)$, we propose to estimate $\alpha_{j}$ and $\beta_{jk}$ by least squares regression of $f_{ij}^{\prime}(t)/f_{ij}(t)$ on $f_{j1}(t)$, …, $f_{jp}(t)$, using data at discrete time points. Considering the large number $p$ of taxa, it is preferable to use regularization estimation methods such as the lasso, ridge, or elastic net regression [39–41]. The estimation procedure is implemented using the **glmnet** function from the **glmnet** R package (v4.1–7) [42]. We denote the estimates of growth rates and interaction parameters by $\hat{\alpha}_{j}$ and $\hat{\beta}_{jk}$, respectively.

### Driver prediction

In order to quantify the contribution of each species in the microbial community, we introduce a novel measure called ‘Driver score’. The driver score is an index based on the concept of a steady state. The steady state refers to a state where the abundances of species no longer change over time (Fig. 1C). Denote by $\alpha$ and $\beta$ the vector of growth rates and the matrix of intensity parameters. The mathematical expression for the steady state can be derived from the gLV equations as ${f}^{\ast }=-{\beta}^{-1}\alpha$.

Loosely speaking, the driver score of a microbe measures the change in steady state before and after perturbation with this microbe (Fig. 1C). Specifically, we start from the community composition in a steady state, denoted by${f}_1^{\ast }=-{\beta}_1^{-1}{\alpha}_1$. We then do a thought experiment by intervening one species, which leads to a new steady state, denoted by ${f}_2^{\ast }=-{\beta}_2^{-1}{\alpha}_2$. The driver score for this species is defined as:


(4) \begin{equation*} D={\left\Vert{f}_2^{\ast }-{f}_1^{\ast}\right\Vert}_2^2, \end{equation*}

where the notation ${\left\Vert \cdotp \right\Vert}_2$ is the Euclidean norm of a vector. This index enables us to evaluate the impact and contribution of each species to the steady state of the microbial community. It is important to note that the driver score reflects a microbe’s influence on the community at a steady state, rather than over different time scales.

**Figure 2:**
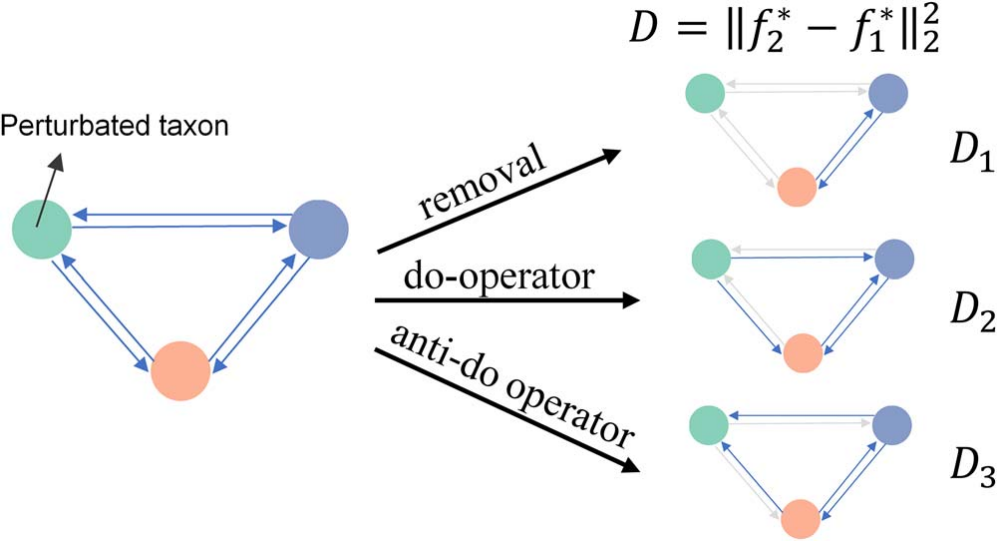
Indices for identifying driver microbes based on community stability. Illustration of the derivation of the ${D}_1$, ${D}_2$, and ${D}_3$ indices by manipulating the causal graph implied by the gLV equations.

We propose three indices to quantify the impact of a particular microbe on community stability (Fig. 2). The first index, ${D}_1$, is determined by the removal of the intervened species from the community. We achieve this by simply zeroing out the corresponding row and column in the intensity matrix ${\beta}_1$, defining ${\beta}_2$to be the modified matrix, while keeping the growth rate vector unchanged. The ${D}_2$ index operates under the assumption that the intervened species can influence other species but is not influenced by them. It is computed by zeroing out the corresponding row in ${\beta}_1$ and setting ${\alpha}_2={\alpha}_1$. In a causal graph perspective, ${D}_2$ represents the outcome of applying the classical do-operator to the ecological network implied by the gLV equations. Conversely, the ${D}_3$ index assumes that the intervened species cannot affect other species but can be influenced by them. This index is determined by zeroing out the corresponding column in ${\beta}_1$ and setting ${\alpha}_2={\alpha}_1$. This action aligns with the concept of performing the anti-do operator on the causal graph. For each index, species can be ranked based on their scores.

### Validation of the three indices using a toy example

**Figure 3:**
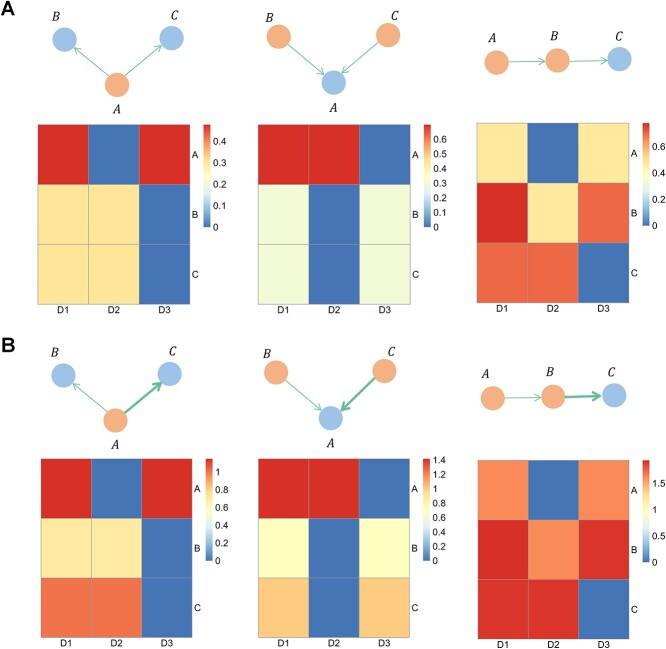
Comparison of ${D}_1$, ${D}_2$, and ${D}_3$ under three simplest causal graph structures: Fork, collider, and chain. In each graph, the orange nodes represent driver microbes, and the thickness of the edges indicates the strength of interactions between microbes. **A** Heatmaps of ${D}_1$, ${D}_2$, and ${D}_3$ with unit intensity of interactions. **B** Heatmaps of ${D}_1$, ${D}_2$, and ${D}_3$ with varying magnitudes of interaction intensities.

In order to gain a first glimpse of the validity of the three indices (${D}_1$, ${D}_2$, and ${D}_3$), we conducted an analysis using three simplest causal graph structures: fork, collider, and chain. The results, shown in Fig. 3A, indicate only the ${D}_3$ index was able to accurately reflect our intuitive understanding of a driver. Figure 3B illustrates the impact of the interaction intensity on each of the indices. We see that increasing the interaction led to an increase in the score, but the validity of ${D}_3$as a driver score was not affected. The toy analysis demonstrates that the ${D}_3$ index had the potential to be useful for driver identification. Therefore, when dealing with real data, ${D}_3$ was chosen as the driver score. Please note that, from a control theory perspective, species with an in-degree of zero—meaning they cannot be influenced by other species—must be controlled to ensure the controllability of the entire system [13]. Clearly, the ${D}_3$ index is distinct from the in-degree metric.

### Simulated data generation

Time-series abundance data were generated based on the negative-binomial distribution and the gLV model in two steps. In the first step, time-series biological signals for each subject were generated from the gLV equations (Eq. (1)), using the **ODE** function of the **deSolve** R package (v1.35) [43].

The parameter setting was as follows. The growth rates ${\alpha}_j$ were drawn from a uniform distribution on the interval$\left(0,0.2\right)$, and for $j\ne k$, the intensity parameters ${\beta}_{jk}$ were sampled from a mixture of a uniform distribution on the interval $\left(-0.0005,0.0005\right)$ with probability $\pi,$ and a degenerate distribution at 0 with probability $1-\pi$. Both the sparse $\pi =0.8$and dense $\pi =0.2$ scenarios were explored. For$j=k$, we set ${\beta}_{jj}=-0.001.$ Finally, the initial abundances were generated from a uniform distribution on the interval $\left(100,10\ 000\right)$.

In the second step, noise abundance data were sample from negative binomial distribution (Eq. (2)), using the **rnegbin** function from the **MASS** R package (v7.3–58.1) [44]. We set the dispersion parameter $\varphi$to take values from the set$\left\{1,3,5\right\}$, with each value representing a different level of noise.

We considered a scenario in which there were 10, 15, or 20 species in the microbial community and 10, 15, or 20 subjects. To mimic real-world situations, we selected varying numbers of time points and intervals for each subject. Specifically, we explored 8, 13, 18, and 25 time points, with random time intervals of 1–5 units.

### Real data applications

To illustrate the application of mbDriver, we utilized two time-series microbiome datasets [45, 46], each comprising a minimum of five time points. One dataset offered absolute abundance data directly, while the other dataset included both 16S rRNA sequencing data and qPCR data. Further details about these two datasets can be found in the **Results** section.

### Bioinformatics and statistical analysis

Two α-diversity indices, Shannon diversity and Simpson diversity, were used to evaluate the evenness and richness of species in a microbial community. Wilcoxon rank sum test was employed to compare the α-diversity among groups. β-diversity based on Bray–Curtis dissimilarity and Principal Coordinate Analysis (PCoA), was used to describe the differences in microbial community composition between groups. Additionally, the statistical significance of β-diversity was analyzed using permutational multivariate analysis of variance (PERMANOVA). The analysis of α-diversity and β-diversity was performed using the **vegan** R package (v2.6–4).

PICRUSt2 (v2.3.0-b), based on the Kyoto Encyclopedia of Genes and Genomes (KEGG) database, was utilized to predict the phylogenetic investigation of microbial communities’ functional profiles [47, 48]. Kruskal–Wallis test was employed for the between-group comparisons of the expressions of level 2 KEGG pathways. Additionally, a linear mixed-effects model was applied to linking metabolites or KEGG pathways with the driver microbes, using the **lmer** function from the **lmerTest** R package (v3.1–3) [49]. The visualization of results was based on the **ggplot2** R package (v3.4.2).

## Results

### Performance of parameter estimation on simulated data

**Figure 4:**
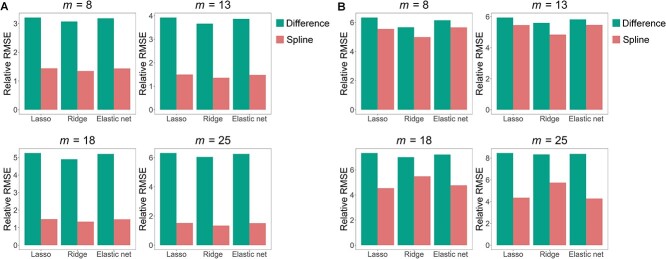
Performance of spline-based and difference-based regularized least squares on simulated data with $n=10$, $p=10$, and dispersion parameter $\varphi =1$. **A** relative root mean squared error (RMSE) of estimates of interaction parameters under a sparse interaction matrix scenario. **B** relative RMSE of estimates of interaction parameters under a dense interaction matrix scenario. $m$ represents the number of time points per subject.

Time-series abundance data were generated using the gLV-based negative-binomial model. Two scenarios were considered: one with a sparse interaction matrix and the other with a dense interaction matrix, each with varying numbers of time points. We preprocessed the simulated data using either a difference-based or spline-based method, and then estimated both interaction and growth rate parameters by applying one of three regularization methods: the lasso, ridge, and elastic net regression. The results, based on 100 replications, are shown in Fig. 4 and Supplementary Figs. 1–4. We observe that under different number of subjects ($n=10,15,20$), and different number of microbes ($p=10,15,20$), the spline-based approach consistently outperformed the difference-based approach, especially when the number of time points was large. This improvement in performance can be attributed to the ability of the negative binomial distribution to effectively capture the overdispersion that is often present in microbiome abundance data. On the other hand, the lasso, ridge, and elastic net regression behaved similarly, and as expected, these methods performed better in the sparse interaction scenario compared to the dense interaction scenario.

**Figure 5:**
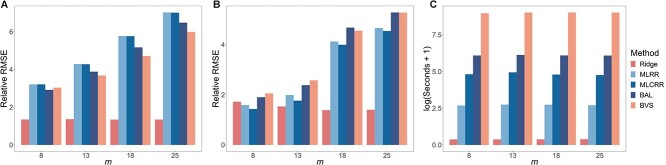
Performance of different estimation methods on simulated data under the sparse interaction matrix scenario with $n=10$, $p=10$, and dispersion parameter $\varphi =1$. **A** relative RMSE for estimates of the interaction matrix. **B** relative RMSE for estimates of the growth rates. **C** the average running time.

Furthermore, we compared our spline and ridge-based method with four methods proposed by Vanni Bucci et al. [27], including differential-based maximum-likelihood ridge regression (MLRR) and maximum-likelihood constrained ridge regression (MLCRR), and spline-based Bayesian adaptive lasso (BAL) and Bayesian variable selection (BVS). Figure 5A, B and Supplementary Fig. 5 demonstrate that, across various parameter settings, our method had the lowest average estimation error for the interaction and growth rates, outperforming its four competitors, which exhibited significantly higher sensitivity to the number of time points. In addition, performance comparisons in predicting temporal profiles, as shown in Supplementary Figs. 6 and 7, reveal that our method had lower prediction error across different parameter settings. On the other hand, our method was computationally the most efficient, as displayed in Fig. 5C. We set the combination of spline and ridge regression as the default choice in mbDriver.

### Application of mbDriver to the dietary fiber intervention dataset

Dietary fiber plays a crucial role in influencing the gut microbiota, which in turn impacts pathophysiology of inflammatory diseases, metabolic syndrome, and obesity [50]. Previous studies have shown that some dietary fibers can be degraded by gut bacteria in the cecum/colon, leading to the production of short-chain fatty acids (SCFAs) [51–53].

**Figure 6:**
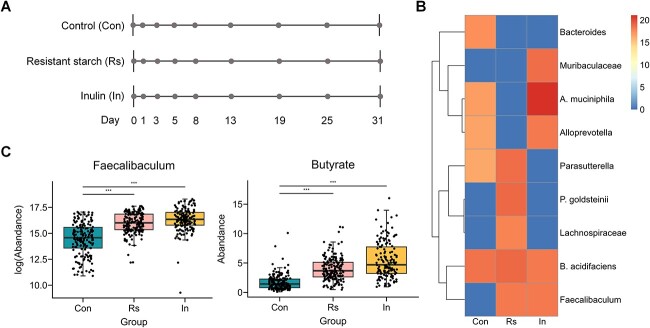
Analysis of microbiome abundance data in a dietary fiber intervention study. **A** description of the time-series dataset. **B** Heatmap of identified driver microbes. **C** boxplots of the abundances of *Faecalibaculum* and butyrate across groups. *** means P-value <0.001.

mbDriver was applied to a dietary fiber intervention dataset, which consists of 508 samples, with 304 microbial annotations at the species level [45]. The dataset was acquired from a time series microbiome study involving three groups of mice that participated in a 31-day experiment (Fig. 6A). One group served as the healthy control (Con), while the other two groups were fed two types of dietary fiber: inulin (In) and resistant starch obtained from corn (Rs).

To assess the α-diversity of the gut microbiota across different groups, the Shannon index and Simpson index were calculated and displayed in Supplementary Fig. 8A, B. We can see that both the richness and evenness of species were significantly lower in the groups that underwent dietary fiber intervention compared to the control group (P-values <0.05). Then, β-diversity analysis based on the Bray–Curtis dissimilarity was performed to gain insights into how microbial communities differ in terms of their composition across different groups, and PCoA was used for data visualization. Supplementary Fig. 8C shows there were significant differences in the composition among the three groups (PERMANOVA, *P* = 0.001).

#### Prediction of driver microbes

To predict the driver microbes in the dietary fiber intervention dataset, we first conducted preprocessing to select species present in more than 80% of the samples, resulting in a total of 37 species. Next, we calculated the sum of species absolute abundances across all samples, focusing on the 10 most abundant species. Finally, the ${D}_3$ index was computed separately for each group. The results are shown in Fig. 6B. In each group, five species with the highest scores were selected as potential drivers. The driver species in the Rs group were *Bacteroides acidifaciens, Parabacteroides goldsteinii, Parasutterella, Faecalibaculum,* and *Lachnospiraceae*. The abundances of these bacteria increased compared to the control group (Supplementary Fig. 9A), and notably, *Parasutterella* and *Faecalibaculum* show significant differences (P-values <0.05). Similarly, the driver species in the In group included *Akkermansia muciniphila, Muribaculaceae, Faecalibaculum, B. acidifaciens,* and *Alloprevotella*. The abundances of these bacteria all increased significantly compared to the control group (P-values <0.05) (Supplementary Fig. 9B). Interestingly, *B. acidifaciens* and *Faecalibaculum* were identified as driver species in both the Rs and In groups. This aligns with reports identifying *B. acidifaciens* and *Faecalibaculum* as primary degraders of inulin, with *Faecalibaculum* also serving as a primary degrader of resistant starch [45]. These primary degraders hydrolyze complex polysaccharide fibers, releasing partial breakdown products (e.g. mono- and oligosaccharides) and fermentation metabolites (e.g. pyruvate) into the gut. This process benefits the secondary fiber degraders and SCFA producers, potentially leading to rapid changes in the gut microbial biomass and SCFA levels [54, 55]. The abundances of *Faecalibaculum* and butyrate, across groups are displayed in Fig. 6C, with their temporal variations shown in Supplementary Fig. 10A, B. We can see that the abundances were significantly higher in the dietary fiber interference group compared to the control group (P-values <0.05). On the other hand, the species that contributed to the stability of the community in the control group were *B. acidifaciens, Bacteroides, A. muciniphila, Alloprevotella,* and *Parasutterella.*

We also calculated the driver score of the top 15 or 20 species with the highest abundance. Each group then selects the top half species with the highest scores as potential drivers. As shown in Supplementary Fig. 11A, B, most of the driver microbes identified using the top 10 species with the highest abundance were also identified using the top 15 or 20 species with the highest abundance, suggesting that increasing the number of candidate species does not have a marked effect on the performance.

#### Correlations between driver microbes and SCFAs

To validate the driver microbes identified by mbDriver, we performed additional analysis using time-series abundance data for six SCFAs, namely acetate, propionate, butyrate, valerate, iso-butyrate, and iso-valerate. A linear mixed-effects model was applied to linking each SCFA with the driver microbes. Specifically, in this model the metabolite was the dependent variable, the driver microbes were the fixed effects, and individuals were treated as random effects. The symbolic description of the model is:


$$ \mathrm{Metabolite}\sim \mathrm{Microbes}+\left(1|\mathrm{Subject}\right). $$

The objective of this analysis was to investigate the contribution of the driver microbes in explaining the observed variability of metabolite levels. Note that we log-transformed microbial abundances after adding a pseudo-count of one to avoid logarithms for zeros.

**Figure 7:**
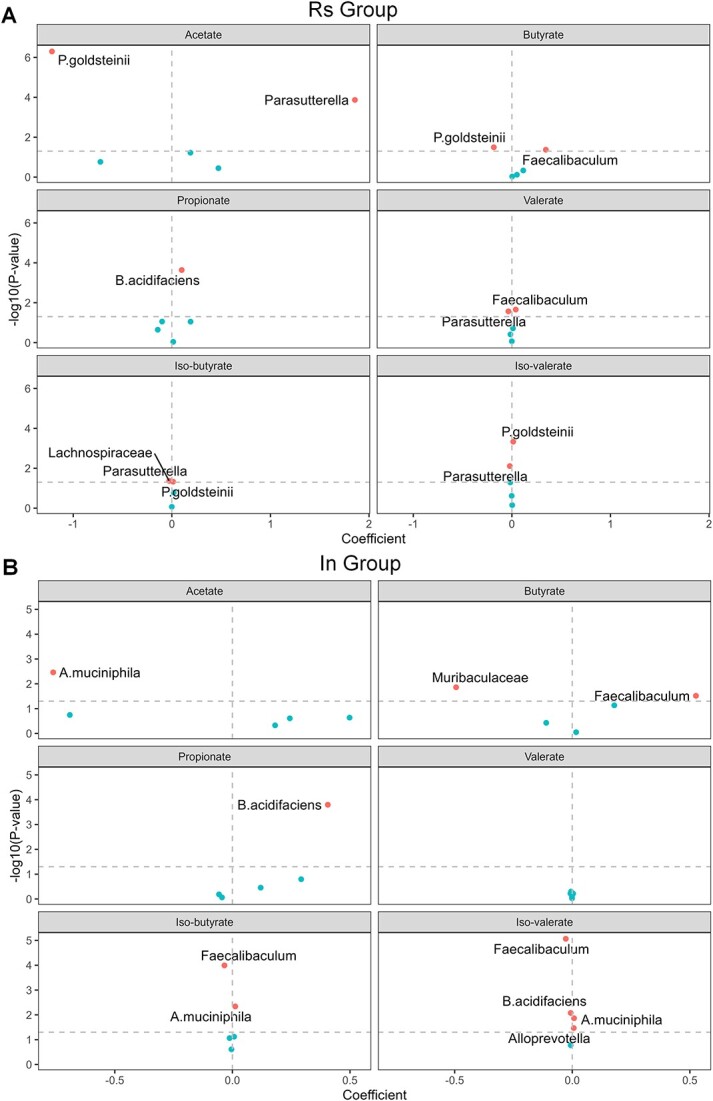
Correlations between driver microbes and SCFAs for the dietary fiber intervention dataset. Visualization of the estimated coefficients of the linear mixed-effects model, linking each of the six SCFAs (acetate, propionate, butyrate, valerate, iso-butyrate, and iso-valerate) with the driver microbes identified in the Rs group **(A)** and the In group **(B)**. The x-axis represents estimated coefficients of the linear mixed-effects model.

In the Rs group, our analysis revealed significant effects of driver microbes on metabolite levels (Fig. 7A). We see that *Faecalibaculum* was beneficial to the production of butyrate and valerate, *P. goldsteinii* was involved in the generation of iso-butyrate and iso-valerate, *Parasutterella* played a role in the production of acetate, and *B. acidifaciens* was linked to the production of propionate. Furthermore, when the total abundance of metabolites was considered as the response variable, *Parasutterella* and *B. acidifaciens* exhibited significant promoting effects (Supplementary Table 1). In addition, we observed significant negative correlations between certain bacteria and short-chain fatty acids, including *P. goldsteinii* with acetate and butyrate, *Parasutterella* with valerate, iso-butyrate, and iso-valerate, as well as *Lachnospiraceae* with iso-butyrate. Previous studies have highlighted the ability of gut microbiota to ferment dietary fiber and produce SCFAs, such as acetate, butyrate, and propionate [53, 56], and notably, *Faecalibaculum* and *B. acidifaciens* are recognized as primary degraders of resistant starch and inulin, facilitating the production of SCFAs [45, 54, 55].

The results for the inulin intervention group are shown in Fig. 7B. *Faecalibaculum* was linked to the production of butyrate, *A. muciniphila* was involved in generation of iso-butyrate and iso-valerate, *B. acidifaciens* tended to produce propionate, and *Alloprevotella* was involved in generation of iso-valerate. When the total abundance of metabolites was considered as the response variable, *B. acidifaciens* demonstrated significant promoting effects (Supplementary Table 1). Similarly, in the In group, we also observe significant negative correlations between certain bacteria and SCFAs, including *Faecalibaculum* with iso-butyrate and iso-valerate, *Muribaculaceae* with butyrate, *A. muciniphila* with acetate, and *B. acidifaciens* with iso-valerate.

In conclusion, the results from the analysis of SCFA data show that the driver microbes identified in the dietary fiber intervention groups had significant effects on the abundances of SCFAs.

#### Compared to MDSINE

We applied MDSINE to the dietary fiber interventions dataset. Specifically, we considered the top 10 species with the highest abundance, estimated parameters in the gLV model using spline-based BVS, and then calculated a keystoneness index to quantify the importance of microbes in an ecosystem [27]. We then selected the top half of the microbes based on their keystoneness values as driver microbes. The results are shown in Supplementary Table 2. When comparing the microbes identified using MDSINE to those identified by mbDriver, as shown in Supplementary Fig. 11C, the overlap in the Con, Rs and In groups was 11.1%, 14.3%, and 25%, respectively. It is worth noting that *Faecalibaculum* is recognized in the literature as a primary degrader of resistant starch and inulin [45], but MDSINE did not predict it as a driver microbe in either of the dietary fiber intervention groups. Furthermore, we investigated the impact of driver microbes identified using MDSINE on SCFA abundances. As shown in Supplementary Fig. 12, in the Rs group, only *B. acidifaciens* had a significant impact on acetate and propionate abundances. In the In group, *Muribaculaceae, B. acidifaciens, P. goldsteinii,* and *Bacteroides* were found to be significantly associated with SCFA abundances.

Additionally, we used MDSINE to predict the driver microbes among the top 15 species with the highest abundance and compared them with those identified by mbDriver. As shown in Supplementary Table 3 and Supplementary Fig. 11D, the overlap in the three groups was 14.3%, 11.1%, and 10%, respectively. Clearly, an increase in the number of candidate species does not lead to a larger overlap in the driver microbes identified by the two methods.

### Application of mbDriver to the ulcerative colitis dataset

**Figure 8:**
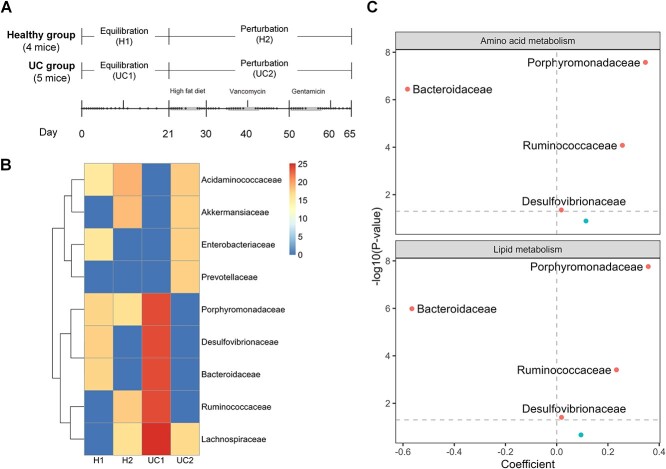
Analysis of microbiome abundance data in an ulcerative colitis study. **A** description of the time-series dataset, with dots representing the sampling points. **B** Heatmap of identified driver microbes. **C** correlation analysis of metabolic pathways and driver microbes of the UC dataset. Visualization of the estimated coefficients of the linear mixed-effects model, linking each of the two UC-associated metabolic pathways (‘Amino acid metabolism’ and ‘lipid metabolism’) with the driver microbes identified in the UC1 group (the disease equilibration period). The x-axis represents estimated coefficients of the linear mixed-effects model.

Ulcerative colitis (UC), with an unknown etiology, is one of the primary forms of inflammatory bowel disease [57]. There has been increasing recognition of the great potential of intestinal microbes as valuable targets for advancing non-invasive strategies in the diagnosis and treatment of UC [58]. Here we acquired both 16S rRNA amplicon sequencing and quantitative PCR data from a high-time resolution perturbation study [46], where samples were collected from mice colonized with human donor flora obtained from a healthy or UC individual. As shown in Fig. 8A, there were five mice in the UC group and four mice in the healthy group, and the experiment extended over a duration of 65 days. The initial 21 days served as an equilibration period, and over the subsequent 44 days mice were exposed to a sequence of three perturbations (high-fat diet, vancomycin, and kanamycin). The dataset consists of 720 samples, with an average of 77 samples per mouse, and 75 microbial annotations at the family level.

The α-diversity of the intestinal microbiota in different groups was measured by calculating the Shannon index and Simpson index. For the healthy group, Supplementary Fig. 13A, B show that both the richness and evenness of microbes were significantly higher in the perturbation group (H2) compared to the equilibration group (H1) (*P* < 0.05). Compared to the healthy perturbation (H2) group, the UC perturbation (UC2) group exhibited significantly lower microbial richness and evenness. Then, β-diversity analysis shows significant differences in gut microbiota composition among these groups (PERMANOVA, *P* = 0.001, Supplementary Fig. 13C).

#### Prediction of driver microbes

We calculated the sum of taxon abundance across all samples, focusing on the 10 most abundant families. Subsequently, we computed the ${D}_3$ index separately for each group. The results are shown in Fig. 8B. In each group, five families with the highest scores were selected as potential drivers. The driver microbes in the H1 group were Desulfovibrionaceae, Bacteroidaceae, Porphyromonadaceae, Enterobacteriaceae, and Acidaminococcaceae. These drivers played a role in maintaining the stability of healthy microbiome composition. After the perturbation, the drivers in the H2 group became Acidaminococcaceae, Akkermansiaceae, Ruminococcaceae, Lachnospiraceae, and Porphyromonadaceae. These bacteria were sensitive to disturbances. On the other hand, in the UC1 group, the driver microbes were Lachnospiraceae, Ruminococcaceae, Porphyromonadaceae, Desulfovibrionaceae, and Bacteroidaceae. These microbes were likely influential in shaping the in the microbial community associated with the UC disease state. Both Lachnospiraceae and Ruminococcaceae have been reported to be associated with UC [58, 59]. In the UC2 group, the drivers responded to the interference conditions were Acidaminococcaceae, Akkermansiaceae, Prevotellaceae, Enterobacteriaceae, and Lachnospiraceae. Recent research has demonstrated that colitis in mice is highly responsive to antibiotics, which can lead to changes in the abundance of Prevotellaceae, Lachnospiraceae, and Enterobacteriaceae [60].

We also calculated the driver scores for the top 15 families and selected the top half families with the highest scores as potential drivers. Supplementary Fig. 14A illustrates that the identified driver microbes included those found using the top 10 families with the highest abundance.

#### Correlation analysis of metabolic pathways and driver microbes

UC is an autoimmune disease that is likely to be influenced by various factors such as genetics, dietary habits, the environment, and the patient’s immune function [61, 62]. An imbalance in the gut microbiota can disrupt normal intestinal immune function, increasing the susceptibility to the development of UC. The interaction between the gut microbiota and the host often occurs through the metabolites produced by the gut microbiota.

To gain a deeper understanding of the metabolic function of the microbial community, we used the PICRUSt2 pipeline to predict the metagenome functions of microbiome. Among these functions, two metabolism-related pathways, ‘Amino acid metabolism’ and ‘Lipid metabolism’, exhibited significant differences between the disease group and the healthy group (Supplementary Table 4). Moreover, studies have shown that these two pathways may be closely related to UC [63]. To further investigate the relationship between driver microbes and metabolic pathways, we employed a linear mixed-effects model. Specifically, the model used is:


$$ \mathrm{Pathway}\sim \mathrm{Microbes}+\left(1|\mathrm{Subject}\right). $$

where individuals were treated as random effects, driver microbes were the fixed effects, and either the ‘Amino acid metabolism’ or ‘Lipid metabolism’ pathway was selected as the dependent variable. Note that we log-transformed microbial abundances and pathway abundances after adding a pseudo-count of one to avoid logarithms for zeros.

The results, shown in Fig. 8C, indicated that among the driver microbes identified in the UC1 group (the disease equilibration period), Ruminococcaceae, Porphyromonadaceae, Desulfovibrionaceae, and Bacteroidaceae were significantly associated with ‘Amino acid metabolism’ pathway and ‘Lipid metabolism’ pathway (*P* < 0.05). These results are consistent with findings from the existing literature. Specifically, Ruminococcus and Bacteroidaceae have been shown to produce SCFAs, which are pivotal in regulating lipid metabolism and maintaining gut health [64, 65]. Moreover, Desulfovibrionaceae plays a role in the degradation of sulfur-containing amino acids, thereby impacting the amino acid metabolism pathway and releasing hydrogen sulfide, which exerts multifaceted effects on gut health, particularly in inflammatory conditions such as ulcerative colitis [66]. In addition, Porphyromonadaceae is involved in the tryptophan metabolism pathway, suggesting potential implications for gut health and inflammatory processes [67]. Collectively, these findings further corroborate the significant associations observed between the driver microbes and the amino acid and lipid metabolism pathways in the UC1 group.

#### Compared to MDSINE

We applied the MDSINE to identify driver microbes among the top 10 families with the highest abundance in the UC dataset, and the results are shown in Supplementary Table 5. In groups H1, H2, UC1, and UC2, there was a 42.9%, 42.9%, 28.6%, and 12.5% overlap, respectively, with the driver microbes identified by mbDriver (Supplementary Fig. 14B). However, in the UC1 group, commonly UC-associated bacteria such as Ruminococcaceae and Lachnospiraceae were identified as driver microbes by mbDriver, but MDSINE did not predict them as driver microbes. Furthermore, we investigated the relationship between driver microbes identified by MDSINE in the disease group and UC-associated metabolic pathways. As shown in Supplementary Fig. 15, only two microbes, Porphyromonadaceae and Sutterellaceae, are significantly correlated with metabolic pathways.

Additionally, we used MDSINE to predict the driver microbes among the top 15 families with the highest abundance and compared them with those identified by mbDriver. As shown in Supplementary Table 6 and Supplementary Fig. 14C, the overlap in the H1, H2, UC1, and UC2 groups was 18.2%, 27.3%, 33.3%, and 45.5%, respectively. Again, there is no discernible pattern between the number of candidate species and the degree of overlap in driver microbes identified by the two methods.

## Discussion

Identifying microbes that significantly influence the structure and function of microbial communities can provide valuable insights into studying disease etiology and potentially offer therapeutic targets for specific conditions [23, 68]. The proposed method, mbDriver, has two major advantages over existing methods. Firstly, for denoising and smoothing time-series abundance data, the application of smoothing splines based on the negative binomial distribution takes into account the characteristics of observed data. Specifically, the nonparametric spline-based method is anticipated to outperform the traditional differential-based method. Secondly, the driver score is derived by manipulating the ecological network or causal graph implied by the gLV equations. This constitutes a fundamental distinction between our proposed index and other indices.

We have validated that mbDriver performed well in simulation studies, and have applied it to two real microbiome datasets. In the dietary fiber intervention dataset, mbDriver identified *Faecalibaculum* as the driver microbe for the group that underwent dietary fiber perturbation. *Faecalibaculum* has been recognized as a primary degrader of resistant starch and inulin, facilitating the production of SCFAs [45, 54, 55]. This was further confirmed through analyses that integrated SCFA data. These analyses also revealed that other microbes identified by mbDriver, *P. goldsteinii*, *B. acidifaciens*, and *Parasutterella* were significantly associated with the production of SCFAs. In the UC-related dataset, mbDriver predicted Lachnospiraceae and Ruminococcaceae as drivers in the disease group, which are critical bacterial groups associated with UC [58, 59]. Furthermore, considering that UC is associated with gut microbiota metabolism, we delved into the correlation between driver microbes and metabolism pathways. The findings highlighted a strong connection between metabolism-associated pathways and the identified driver microbes, including Ruminococcaceae, Porphyromonadaceae, Desulfovibrionaceae, and Bacteroidaceae. By analyzing these two real datasets, we have demonstrated not only the application of mbDriver but also its effectiveness in predicting driver microbes.

The proposed methodology still has some limitations that need to be addressed. First, the proposed driver score is context-dependent, meaning it relies on the specific community it is applied to. Consequently, it may not be suitable for situations where inter-species interactions undergo significant fluctuations over time. Second, during the data preprocessing step, low-abundance microbes are filtered out to enhance the reliability of the constructed ecological network [69]. However, this has the risk of introducing biases into downstream analyses, since rare species may have a significant impact on the microbial community [70]. Indeed, microbiome sequencing data often exhibit sparsity, with a substantial proportion of zero values. To tackle this issue, an interesting future direction is the extension of the negative binomial distribution to a zero-inflated negative binomial distribution [71]. Moreover, given the prevalence of species data represented in relative abundances, extending the proposed framework from absolute to relative abundances will broaden its applicability. Nevertheless, the application of zero-inflated models can be challenging due to the temporal nature of the data. Third, the current version of mbDriver solely relies on species abundance data. However, for a more comprehensive understanding of the microbiome, simply knowing the composition of a microbial community is insufficient. As demonstrated in the real data application, gaining insights into the community’s functions requires the integration of other types of omics data [18]. Recent research has highlighted that metabolite levels in the gut often hold greater predictive power for host health than species levels [72, 73]. To incorporate time-series metabolomic data into mbDriver, one approach is to consider the interactions between microbes and their metabolites [74]. We are actively working on developments in this direction. Fourth, the classical gLV model and the associated ecological network focus on pairwise interactions. Nevertheless, pairwise modeling may fail to capture diverse pairwise microbial interactions [75], and multiple studies have presented evidence for the existence of higher-order interactions, which involve changes in interactions among a group of species due to the presence of another group of species [76, 77]. Addressing the inclusion of higher-order interactions, both in theory and practice, presents an ongoing challenge [78]. This challenge revolves around understanding and modeling the complex dependencies that emerge when considering the collective influence of multiple species on one another within a microbial community.

While the gLV model is widely used to describe microbial community dynamics, the assumptions underlying this model may be violated in real-world data scenarios, potentially leading to misconceptions about community stability and function. Therefore, it is crucial to recognize the limitations of the gLV model in practical applications, including: (i) parameters may be unidentifiable, particularly in high-dimensional settings, due to insufficient absolute abundance time series data; (ii) the assumption of parameter universality may not hold, as communities in real ecosystems are subject to dynamic changes; and (iii) higher-order interactions may exist, indicating that the interactions between two species could be influenced by the presence of other species [31, 75, 77].

Finally, it is important to emphasize that identifying driver microbes is a formidable task, given the diversity and complexity of microbial communities and the existence of unculturable microbes. mbDriver has been purposefully designed to provide a catalog of candidate drivers, which, to a certain extent, helps alleviate the challenges associated with experimental validation. While mbDriver has been demonstrated to be a reliable screening tool, it remains essential to experimentally validate the driver microbes it identifies.

**Key Points**

- We defined driver microbes as those capable of inducing changes in the community’s steady state and significantly contributing to these changes.
- To identify such driver microbes, we introduced a novel framework called mbDriver, which analyzes microbiome abundance data collected at discrete time points.
- mbDriver consists of data preprocessing using smoothing splines, parameter estimation for the gLV model, and quantification of each microbe’s contribution to the community’s steady state.
- We demonstrated mbDriver’s superior performance on both simulated and real-world datasets. This framework holds promise for studying the dynamics of human microbiota and predicting microbiota-based targets for disease treatment.

## Supplementary Material

Supp_mbDriver_BIB-24-1205_bbae580

Supplementary_table4_bbae580

## Data Availability

We applied mbDriver to two real datasets. The dietary fiber intervention dataset was acquired from a time series microbiome study [45] and the UC dataset was collected from a high-time resolution perturbation study [46]. The source code and data for reproducing main figures in the article are available at https://github.com/tanxiaoxiu/mbDriver.
