# Supplementary material for: mbDriver: identifying driver microbes in microbial communities based on time-series microbiome data
Source: Brief Bioinform. 2024 Nov 11;25(6):bbae580. doi: 10.1093/bib/bbae580 (PMC11551971; doi:10.1093/bib/bbae580)
Supplement: Supp_mbDriver_BIB-24-1205_bbae580 [file supp_mbdriver_bib-24-1205_bbae580.pdf]

Supplementary information

Regarding the time comparison, the device used was Intel(R) Xeon(R) Gold 6240R CPU @ 2.40GHz. For each of MLRR, MLCRR, BAL and BVS, 16 cores were allocated for each task, and for our spline-based ridge method, 1 core was allocated for each task.

Supplementary Figures

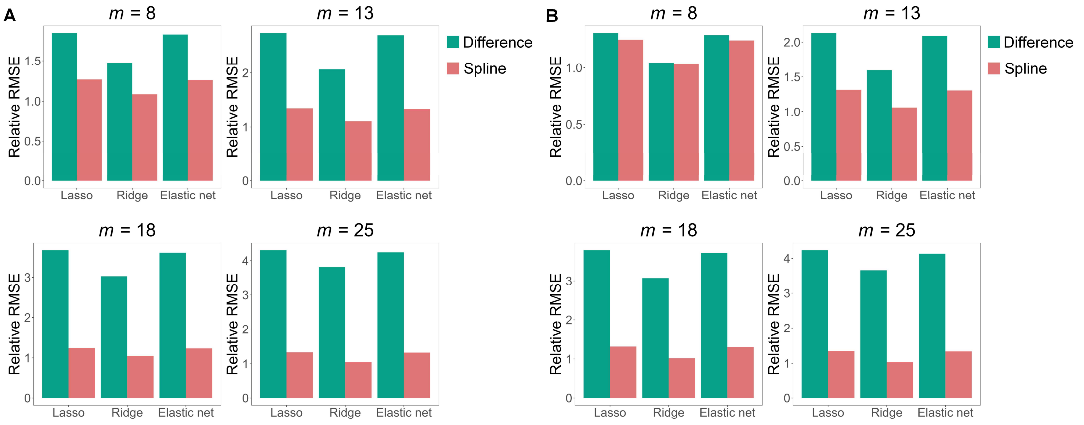

Supplementary Fig. 1: Performance of spline-based and difference-based regularized least squares on simulated data under the sparse interaction matrix scenario, with $n = 10$ subjects and $p = 10$ taxa. **A** RMSE of estimates of interaction parameters with dispersion parameter $\varphi = 3$. **B** Relative RMSE of estimates of interaction parameters with dispersion parameter $\varphi = 5$. m represents the number of time points per subject.

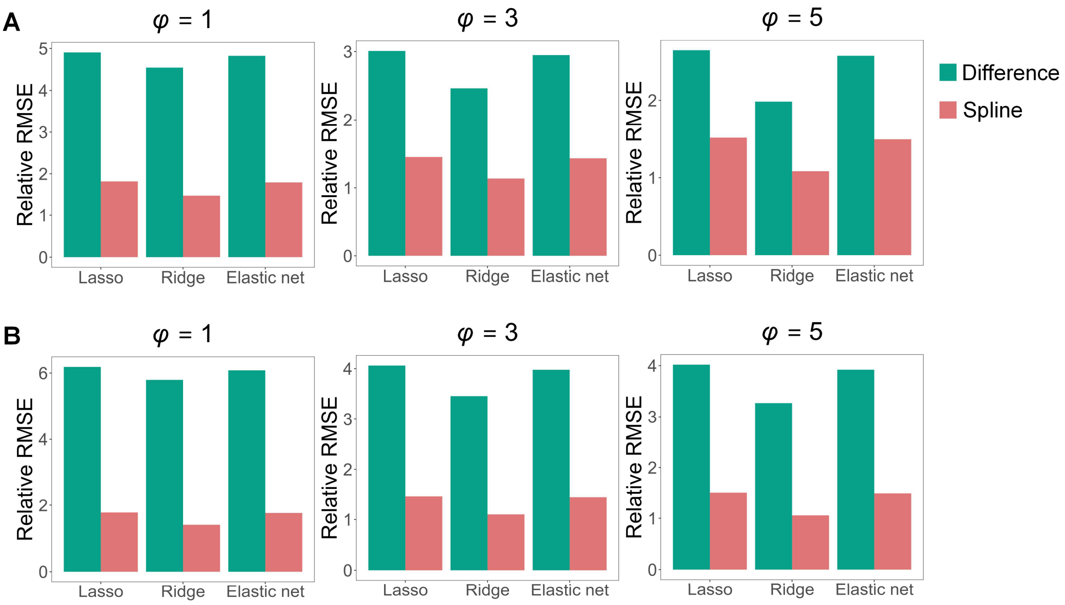

Supplementary Fig. 2: Performance of spline-based and difference-based regularized least squares on simulated data under the sparse interaction matrix scenario, with $n = 10$ subjects and $p = 15$ taxa. **A** RMSE of estimates of interaction parameters with $m = 13$ time points. **B** Relative RMSE

of estimates of interaction parameters with $m = 18$ time points.

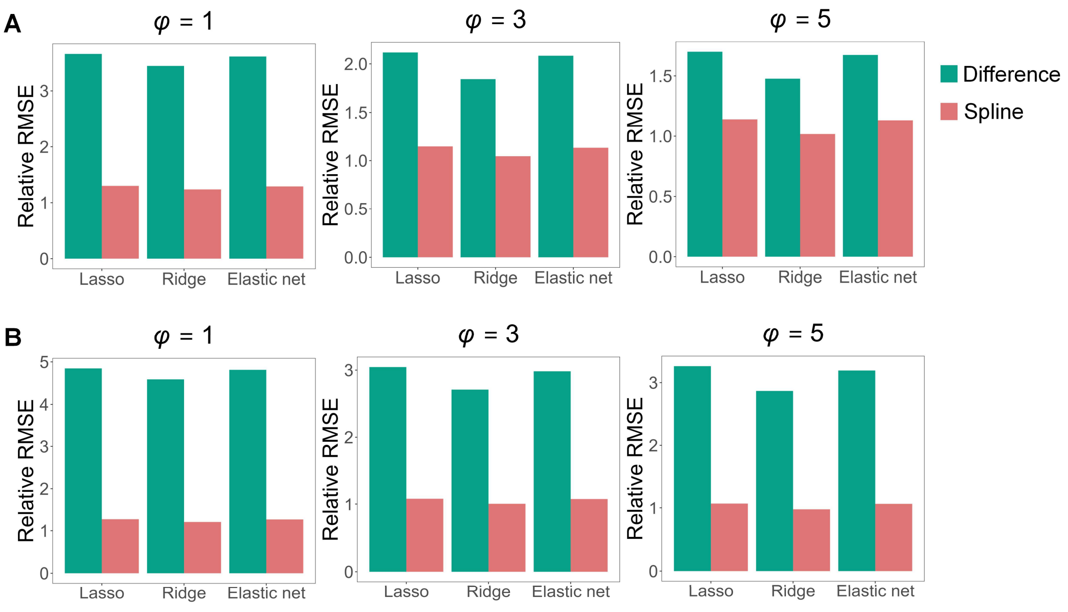

Supplementary Fig. 3: Performance of spline-based and difference-based regularized least squares on simulated data under the sparse interaction matrix scenario, with $n = 15$ subjects and $p = 10$ taxa. **A** RMSE of estimates of interaction parameters with $m = 13$ time points. **B** Relative RMSE of estimates of interaction parameters with $m = 18$ time points.

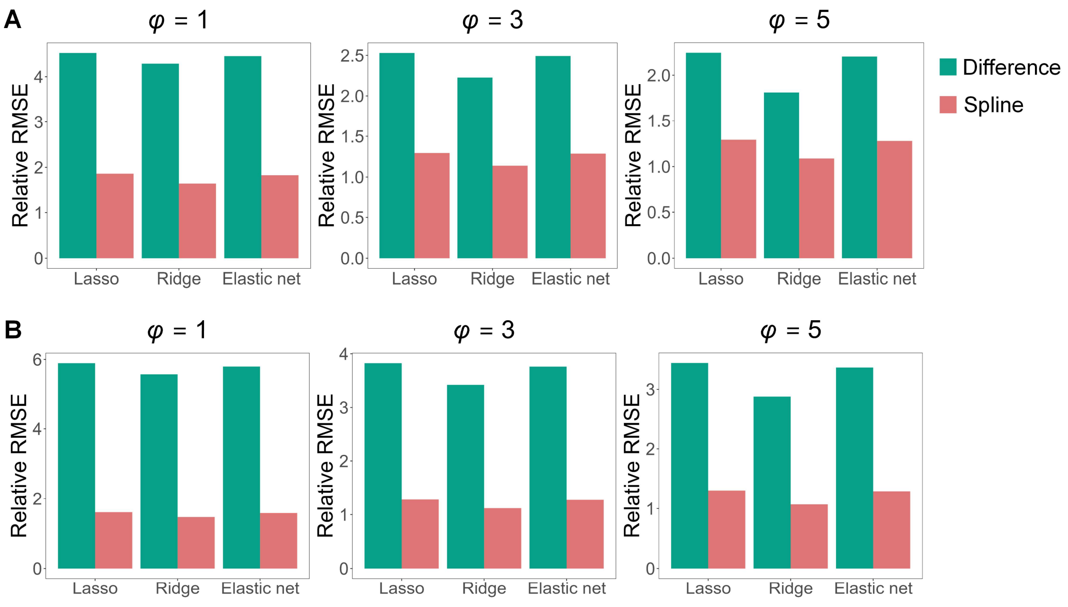

Supplementary Fig. 4: Performance of spline-based and difference-based regularized least squares on simulated data under the sparse interaction matrix scenario, with $n = 20$ subjects and $p = 20$ taxa. **A** RMSE of estimates of interaction parameters with $m = 13$ time points. **B** Relative RMSE of estimates of interaction parameters with $m = 18$ time points.

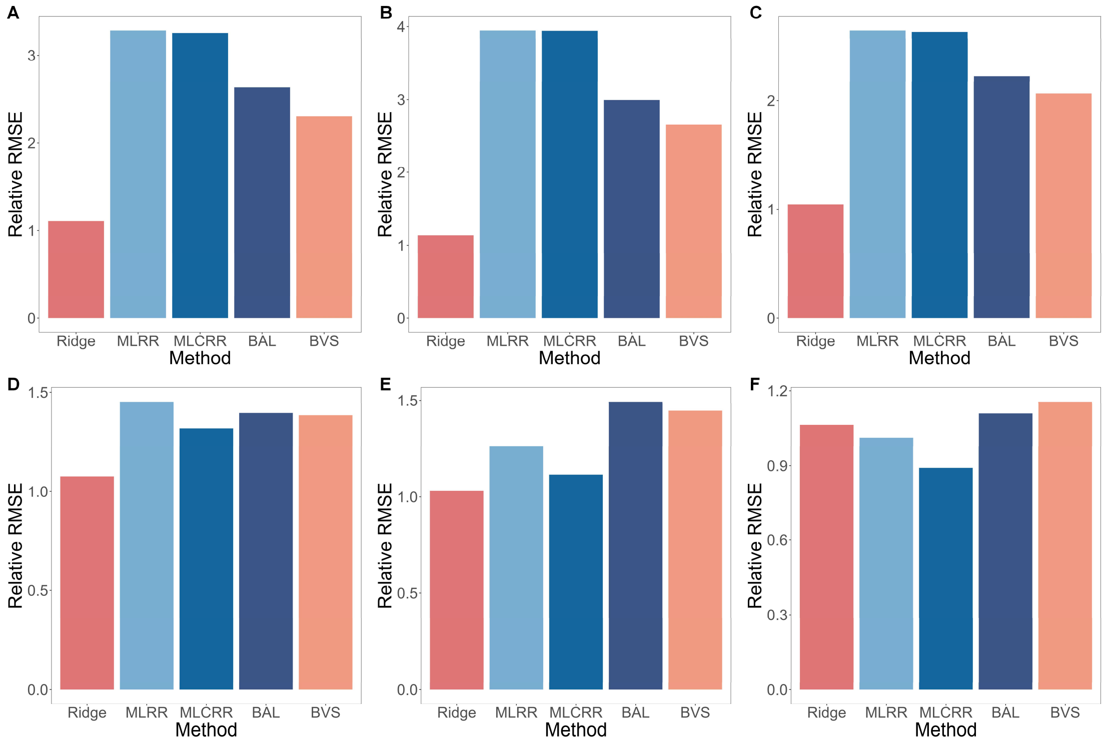

Supplementary Fig. 5: Performance of different estimation methods on simulated data under the sparse interaction matrix scenario, with $m = 13$ time points and dispersion parameter $\varphi = 3$. **A** Relative RMSE for estimates of the interaction matrix with $n = 10$ and $p = 10$. **B** Relative RMSE for estimates of the interaction matrix with $n = 10$ and $p = 15$. **C** Relative RMSE for estimates of the interaction matrix with $n = 15$ and $p = 10$. **D** Relative RMSE for estimates of the growth rates with $n = 10$ and $p = 10$. **E** Relative RMSE for estimates of the growth rates with $n = 10$ and $p = 15$. **F** Relative RMSE for estimates of the growth rates with $n = 15$ and $p = 10$.

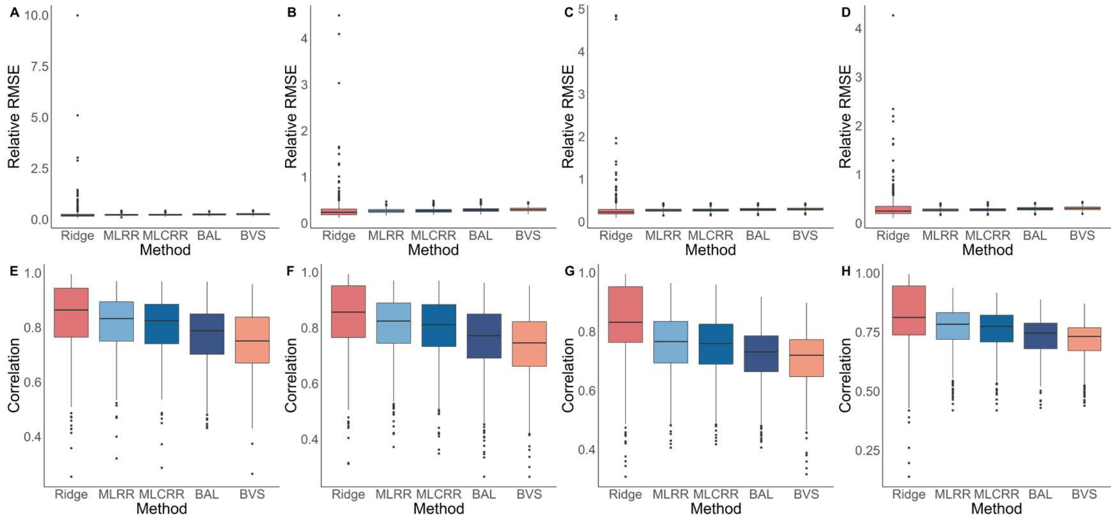

Supplementary Fig. 6: Performance of trajectories predicted by different estimation methods on

simulated data under the sparse interaction matrix scenario with $n = 10$, $p = 10$, and dispersion parameter $\varphi = 1$. **A-D** Relative RMSE for estimates of the trajectories with time points $m = 8, 13, 18, 25$, respectively. **E-H** Pearson correlation between predicted and actual trajectories with time points $m = 8, 13, 18, 25$, respectively.

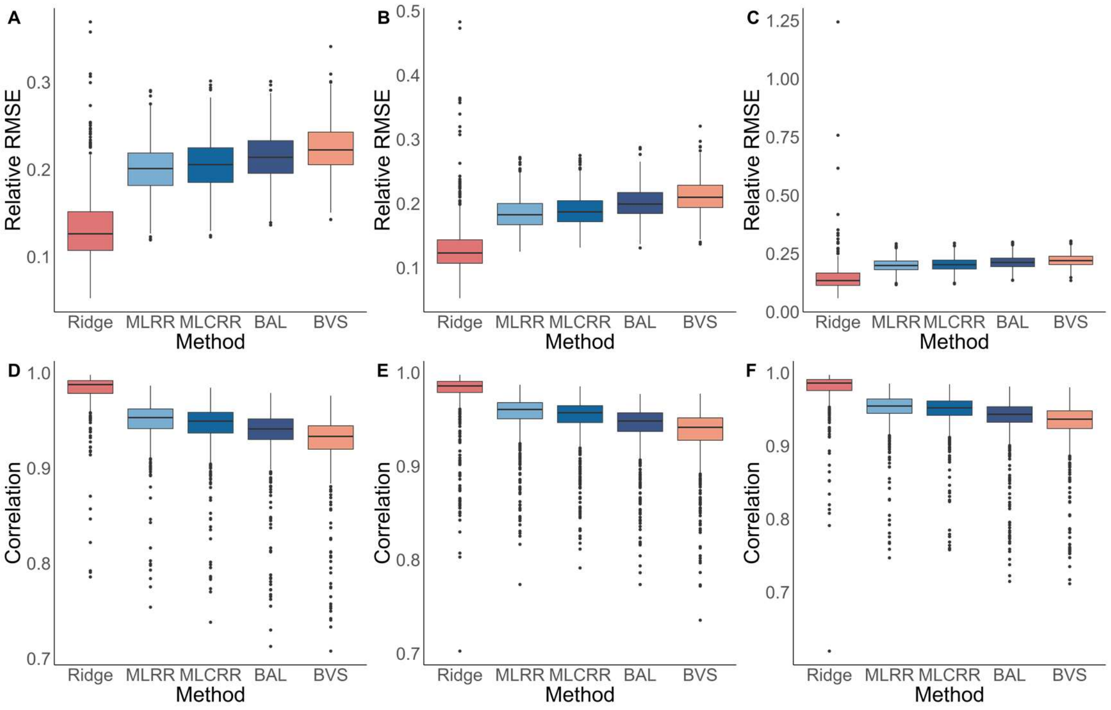

Supplementary Fig. 7: Performance of trajectories predicted by different estimation methods on simulated data under the sparse interaction matrix scenario, with $m = 13$ time points and dispersion parameter $\varphi = 3$. **A** Relative RMSE for estimates of the trajectories with $n = 10$ and $p = 10$. **B** Relative RMSE for estimates of the trajectories with $n = 10$ and $p = 15$. **C** Relative RMSE for estimates of the trajectories with $n = 15$ and $p = 10$. **D** Pearson correlation between predicted and actual trajectories with $n = 10$ and $p = 10$. **E** Pearson correlation between predicted and actual trajectories with $n = 10$ and $p = 15$. **F** Pearson correlation between predicted and actual trajectories with $n = 15$ and $p = 10$.

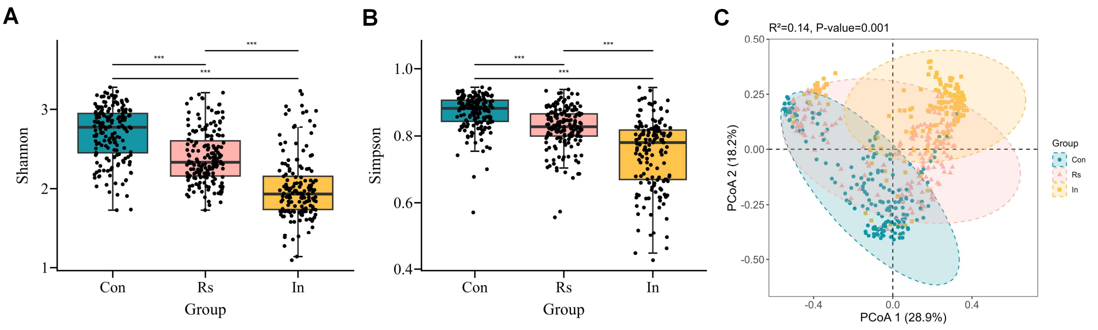

Supplementary Fig. 8: Differences in microbiome composition in the dietary fiber intervention dataset. **A, B** α-diversity analysis based on the Shannon diversity and Simpson diversity. **C** β-

diversity analysis based on the Bray-Curtis distance. *** means P-value < 0.001.

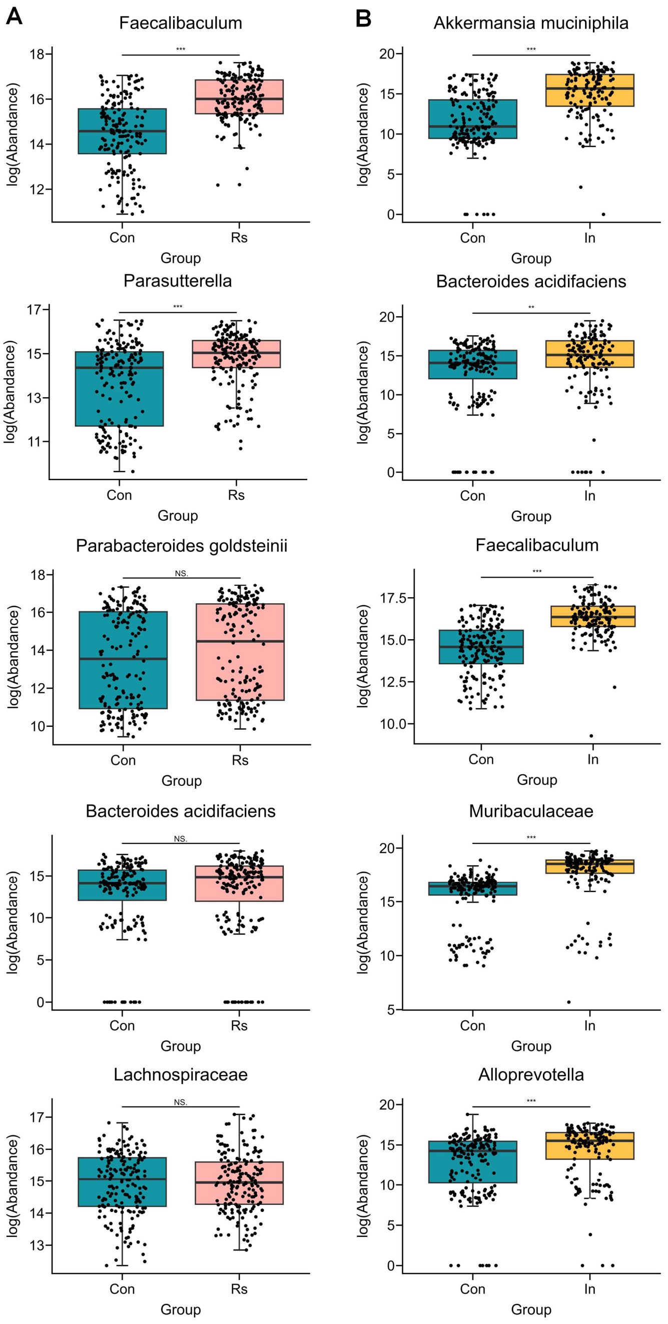

Supplementary Fig. 9: Boxplots of the abundances of driver microbes across groups. NS means not significant, * means P-value < 0.05, ** means P-value < 0.01, and *** means P-value < 0.001.

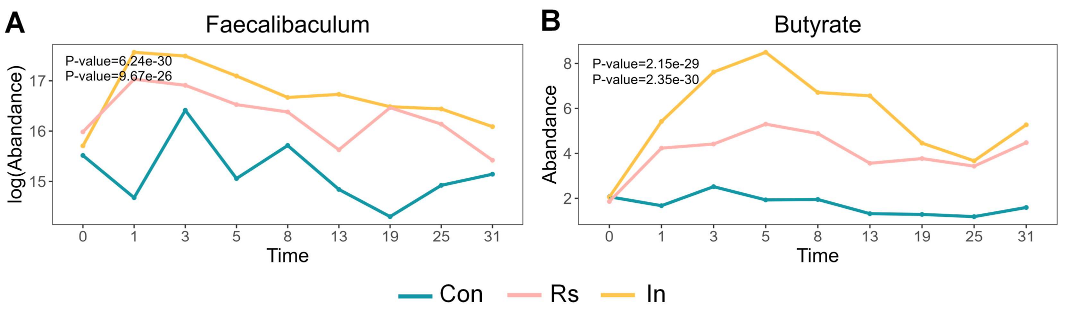

Supplementary Fig. 10: The temporal changes in the abundances of *Faecalibaculum* (A) and butyrate (B) across in the Con, Rs, and In groups.

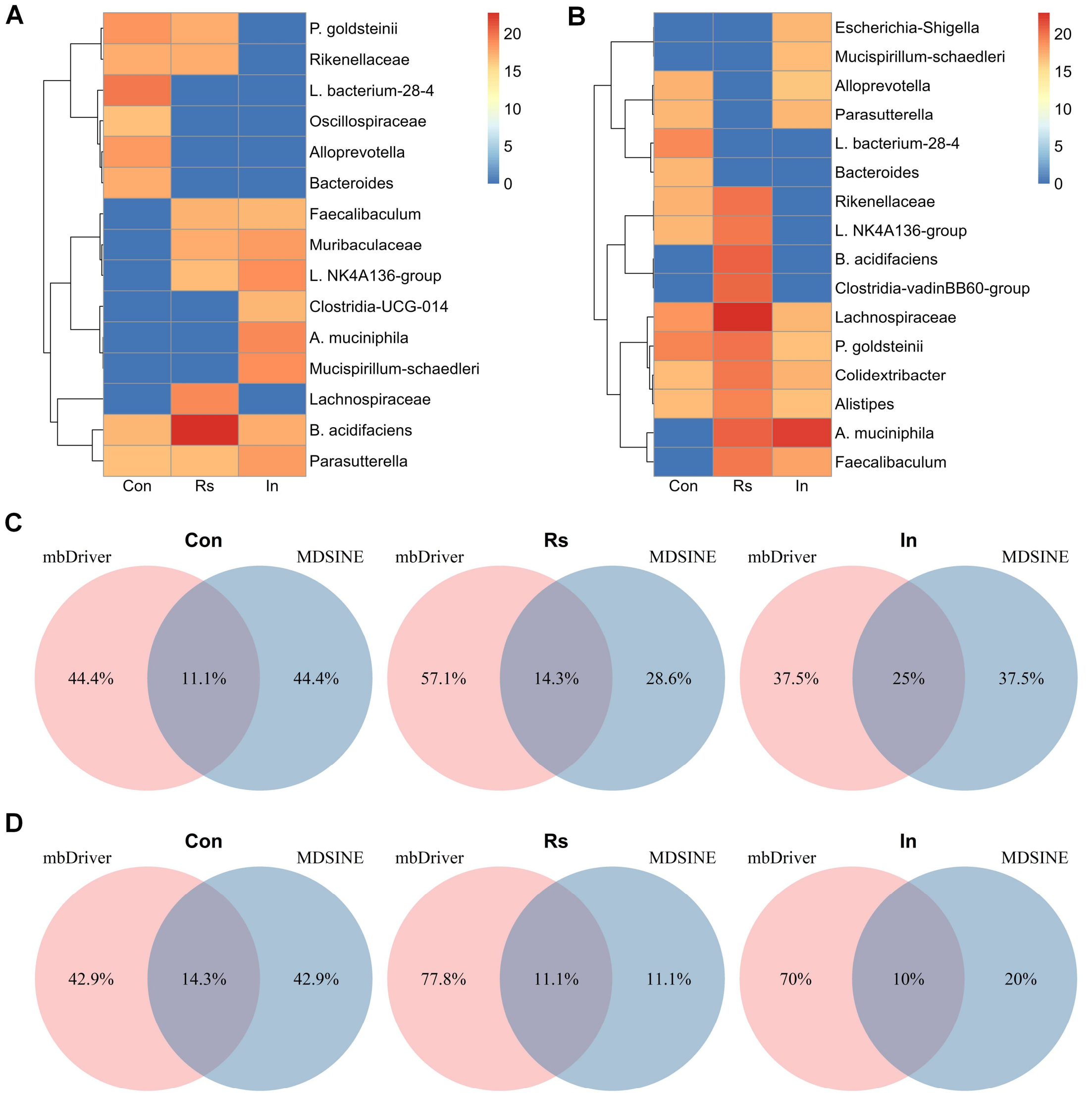

Supplementary Fig. 11: Expanded analysis of driver microbes in the dietary fiber intervention study. **A, B** The driver microbes identified by mbDriver for the top 15 and top 20 species with the

highest abundance, respectively. **C, D** Comparison of driver microbes identified by mbDriver and MDSINE across the Con, Rs, and In groups using the top 10 and top 15 species with the highest abundance, respectively.

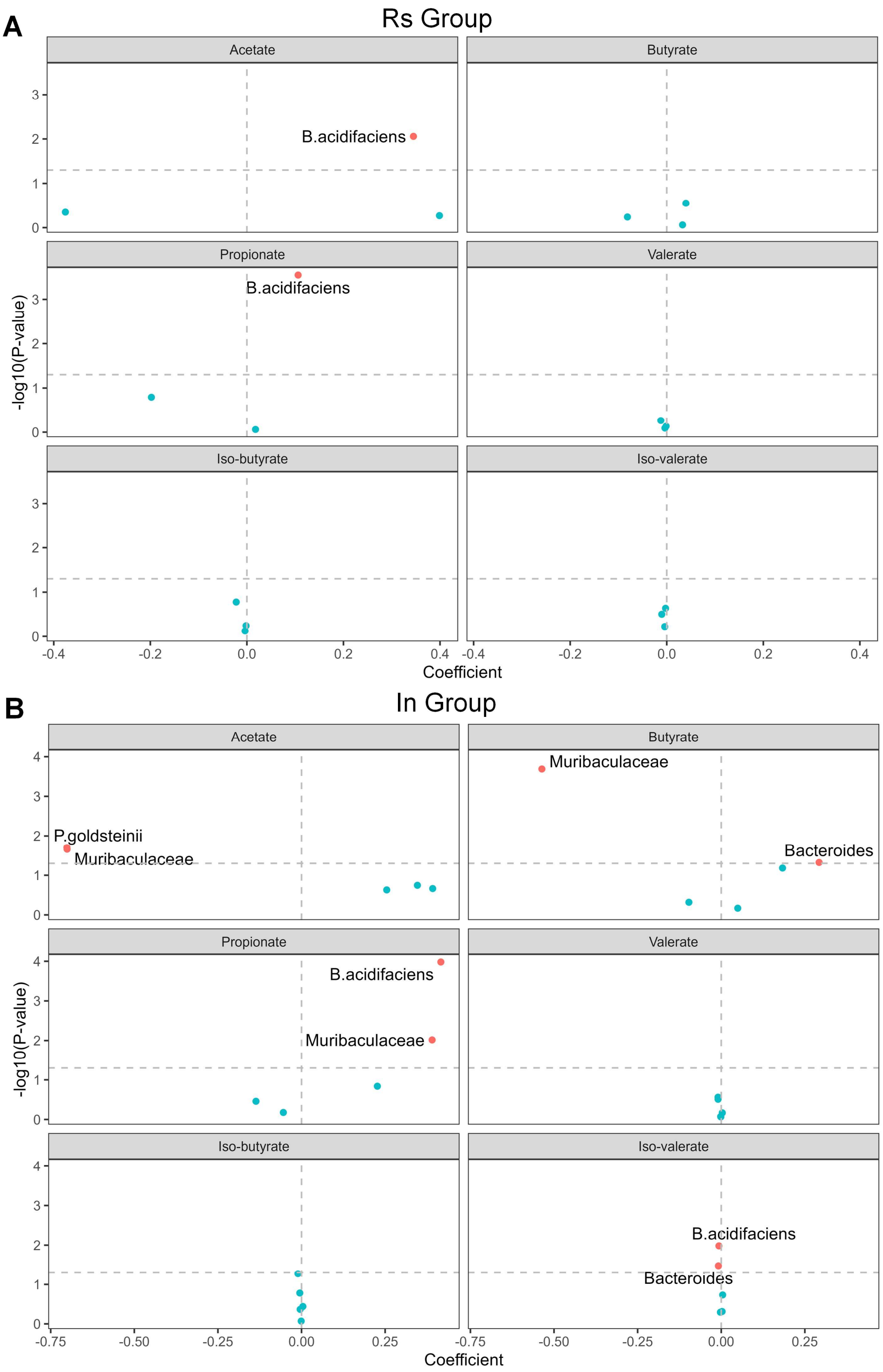

Supplementary Fig. 12: Correlation analysis between SCFAs and driver microbes identified by MDSINE in the dietary fiber intervention dataset. Visualization of the estimated coefficients of the linear mixed-effects model, linking each of the six SCFAs (acetate, propionate, butyrate, valerate,

iso-butyrate, and iso-valerate) with the driver microbes identified in the Rs group **(A)** and the In group **(B)**. The red dots, each labeled with a taxon name, indicate microbes significantly correlated with SCFAs. The x-axis represents estimated coefficients of the linear mixed-effects model.

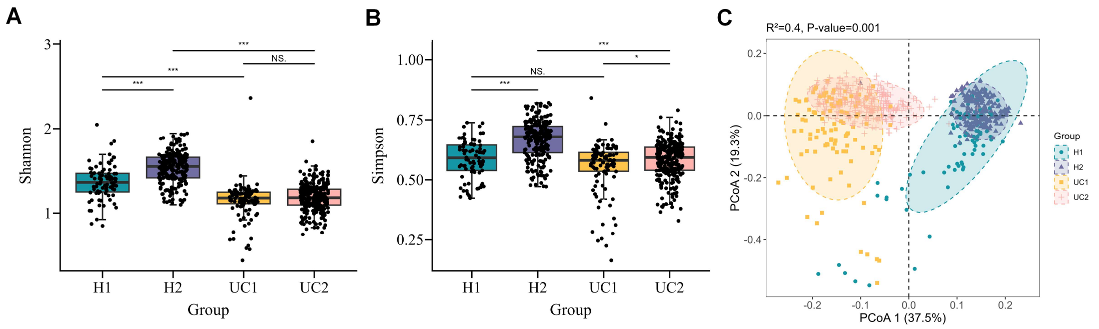

Supplementary Fig. 13: Differences in microbiome composition in the ulcerative colitis dataset. **A, B** α-diversity analysis based on the Shannon diversity and Simpson diversity. **C** β-diversity analysis based on the Bray-Curtis distance. NS means not significant, * means P-value < 0.05, ** means P-value < 0.01, and *** means P-value < 0.001.

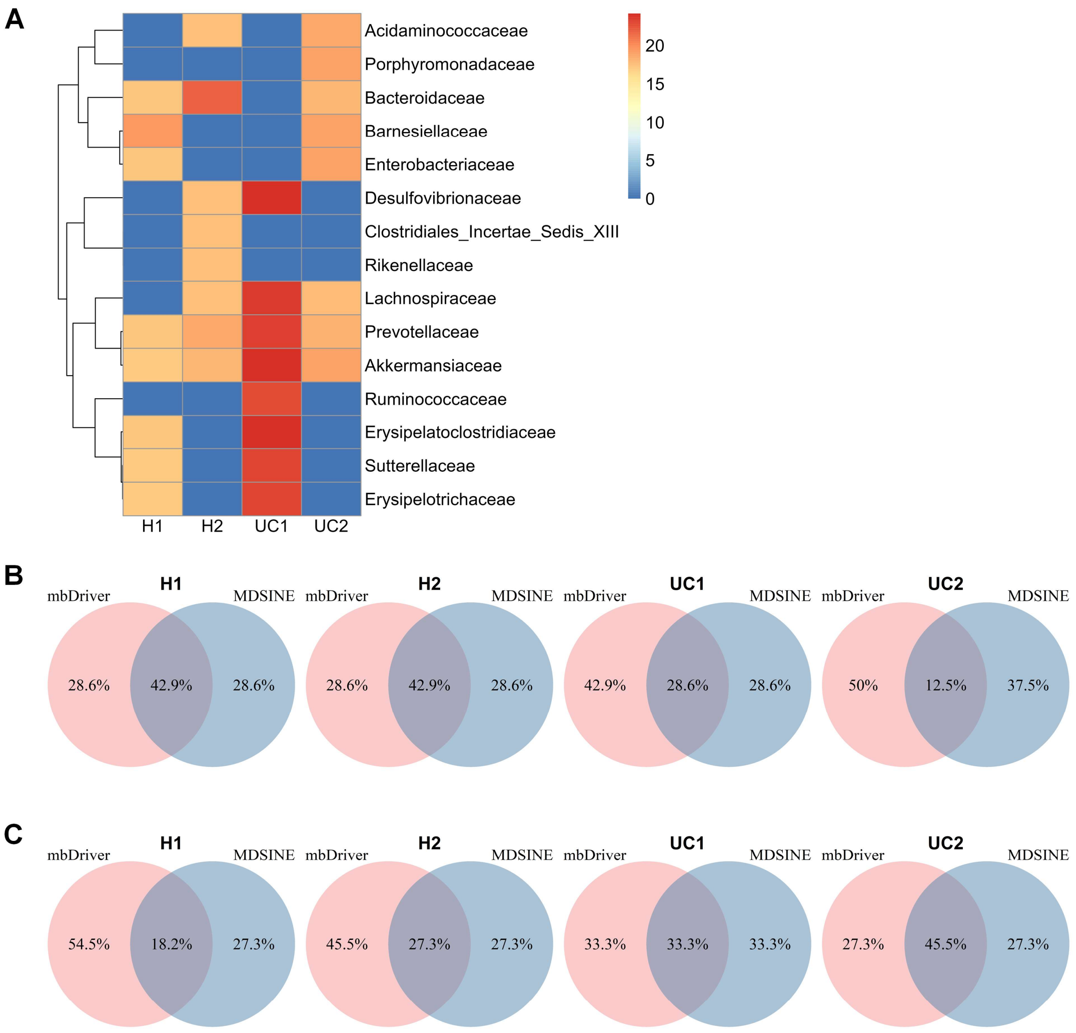

Supplementary Fig. 14: Expanded analysis of driver microbes in the ulcerative colitis dataset. **A** The driver microbes identified by mbDriver for the top 15 families with the highest abundance. **B, C** Comparison of driver microbes identified by mbDriver and MDSINE across the H1, H2, UC1 and UC2 groups using the top 10 and top 15 families with the highest abundance, respectively.

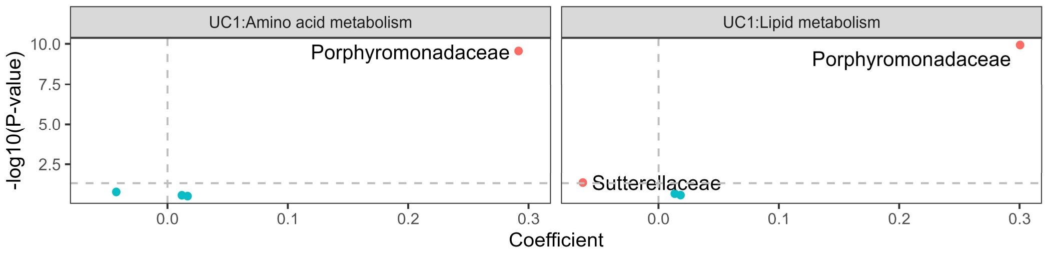

Supplementary Fig. 15: Correlation analysis of metabolic pathways and driver microbes identified by MDSINE in the UC dataset. Visualization of the estimated coefficients of the linear mixed-effects

model, linking each of the two UC-associated metabolic pathways (“Amino acid metabolism” and “Lipid metabolism”) with the driver microbes identified in the UC1 group (the disease equilibration period). The red dots, each labeled with a taxon name, indicate microbes significantly correlated with pathways. The x-axis represents estimated coefficients of the linear mixed-effects model.

Supplementary Tables

Supplementary Table 1: Microbes that have a significant effect on total metabolites.

Group	Microbe	Estimate	Std. Error	df	t value	Pr(>|t|)
	P.goldsteinii	-1.40966	0.328085	177	-4.29663	2.86E-05
**Rs**	Parasutterella	1.960213	0.669822	177	2.926469	0.003878
	B.acidifaciens	0.297377	0.139993	177	2.124227	0.03504
**In**	B.acidifaciens	0.830174	0.359404	151	2.309866	0.02225

Supplementary Table 2: Driver microbes predicted by MDSINE when applied to the top 10 species with the highest abundance from the dietary fiber intervention dataset.

Group	Microbe				
**Con**	P.goldsteinii	L.NK4A136-group	Muribaculaceae	B.acidifaciens	Bacteroides
**Rs**	Lachnospiraceae	B.acidifaciens	L.NK4A136-group	NA	NA
**In**	Muribaculaceae	Alloprevotella	B.acidifaciens	P.goldsteinii	Bacteroides

Supplementary Table 3: Driver microbes predicted by MDSINE when applied to the top 15 species with the highest abundance from the dietary fiber intervention dataset.

**Con**	**Rs**	**In**
Muribaculaceae	Lachnospiraceae	Muribaculaceae
P.goldsteinii	B. acidifaciens	B. acidifaciens
L.NK4A136-group	NA	Alloprevotella
Oscillospiraceae	NA	NA
Lachnospiraceae	NA	NA
Mucispirillum.schaedleri	NA	NA
Parasutterella	NA	NA
Rikenellaceae.RC9.gut.group	NA	NA

Supplementary Table 4: “Amino acid metabolism” and “Lipid metabolism” pathways. See Supplementary Table 4 in Excel format.

Supplementary Table 5: Driver microbes predicted by MDSINE when applied to the top 10 families with the highest abundance from the UC dataset.

Group	Microbe				
**H1**	Enterobacteriaceae	Bacteroidaceae	Akkermansiaceae	Prevotellaceae	Porphyromonadaceae
**H2**	Enterobacteriaceae	Bacteroidaceae	Porphyromonadaceae	Lachnospiraceae	Akkermansiaceae
**UC1**	Porphyromonadaceae	Prevotellaceae	Desulfovibrionaceae	Sutterellaceae	NA
**UC2**	Porphyromonadaceae	Prevotellaceae	Desulfovibrionaceae	Sutterellaceae	NA

Supplementary Table 6: Driver microbes predicted by MDSINE when applied to the top 15 families with the highest abundance from the UC dataset.

**H1**	**H2**	**UC1**	**UC2**
Acidaminococcaceae	Clostridiales_Incertae_Sedis_XIII	Clostridiales_Incertae_Sedis_XIII	Clostridiales_Incertae_Sedis_XIII
Clostridiales_Incertae_Sedis_XIII	Acidaminococcaceae	Porphyromonadaceae	Desulfovibrionaceae
Ruminococcaceae	Ruminococcaceae	Lachnospiraceae	Lachnospiraceae
Sutterellaceae	Sutterellaceae	Prevotellaceae	Porphyromonadaceae
Erysipelotrichaceae	Rikenellaceae	Ruminococcaceae	Prevotellaceae
NA	Erysipelotrichaceae	Barnesiellaceae	Ruminococcaceae
NA	NA	Acidaminococcaceae	Barnesiellaceae
NA	NA	Desulfovibrionaceae	Acidaminococcaceae
